# Sequential Adaptive Mutations Enhance Efficient Vector Switching by Chikungunya Virus and Its Epidemic Emergence

**DOI:** 10.1371/journal.ppat.1002412

**Published:** 2011-12-08

**Authors:** Konstantin A. Tsetsarkin, Scott C. Weaver

**Affiliations:** Institute for Human Infections and Immunity, Center for Tropical Diseases, and Department of Pathology, University of Texas Medical Branch, Galveston, Texas, United States of America; University of North Carolina at Chapel Hill, United States of America

## Abstract

The adaptation of Chikungunya virus (CHIKV) to a new vector, the *Aedes albopictus* mosquito, is a major factor contributing to its ongoing re-emergence in a series of large-scale epidemics of arthritic disease in many parts of the world since 2004. Although the initial step of CHIKV adaptation to *A. albopictus* was determined to involve an A226V amino acid substitution in the E1 envelope glycoprotein that first arose in 2005, little attention has been paid to subsequent CHIKV evolution after this adaptive mutation was convergently selected in several geographic locations. To determine whether selection of second-step adaptive mutations in CHIKV or other arthropod-borne viruses occurs in nature, we tested the effect of an additional envelope glycoprotein amino acid change identified in Kerala, India in 2009. This substitution, E2-L210Q, caused a significant increase in the ability of CHIKV to develop a disseminated infection in *A. albopictus,* but had no effect on CHIKV fitness in the alternative mosquito vector, *A. aegypti,* or in vertebrate cell lines. Using infectious viruses or virus-like replicon particles expressing the E2-210Q and E2-210L residues, we determined that E2-L210Q acts primarily at the level of infection of *A. albopictus* midgut epithelial cells. In addition, we observed that the initial adaptive substitution, E1-A226V, had a significantly stronger effect on CHIKV fitness in *A. albopictus* than E2-L210Q, thus explaining the observed time differences required for selective sweeps of these mutations in nature. These results indicate that the continuous CHIKV circulation in an *A. albopictus*-human cycle since 2005 has resulted in the selection of an additional, second-step mutation that may facilitate even more efficient virus circulation and persistence in endemic areas, further increasing the risk of more severe and expanded CHIK epidemics.

## Author Summary

Since 2004, chikungunya virus (CHIKV) has caused a series of devastating outbreaks in Asia, Africa and Europe that resulted in up to 6.5 million cases of arthritic disease and have been associated with several thousand human deaths. Although the initial step of CHIKV adaptation to *A. albopictus* mosquitoes, which promoted re-emergence of the virus, was determined to involve an E1-A226V amino acid substitution, little attention has been paid to subsequent CHIKV evolution after this adaptive mutation was convergently selected in several geographic locations. Here we showed that novel substitution, E2-L210Q identified in Kerala, India in 2009, caused a significant increase in the ability of CHIKV to infect and develop a disseminated infection in *A. albopictus.* This may facilitate even more efficient virus circulation and persistence in endemic areas, further increasing the risk of more severe and expanded CHIK epidemics. Our findings represent some of the first evidence supporting the hypothesis that adaptation of CHIKV (and possible other arboviruses) to new niches is a sequential multistep process that involves selections of at least two adaptive mutations.

## Introduction

The potential of RNA viruses to emerge into new environments often depends on their ability to efficiently adapt to new hosts. These adaptations sometimes comprise a stepwise process that includes 1) initial viral introduction/establishment in the recipient species, followed by 2) finite adjustment/optimization of the virus replication and transmission strategies for specific environments associated with a new host [1], [2]. This process has been well documented for several single-host viruses such as pandemic influenza A virus, the SARS coronavirus and canine parvovirus (reviewed in [3], [4]) that do not rely on alternating infection of disparate hosts for their maintenance in nature. However, much less is known about the adaptive processes that mediate cross-species jumps for double-host viruses such as arthropod-borne viruses (arboviruses). Several recent studies documented that the acquisition of a single mutation in an arbovirus genome can mediate their cross-species transfer [step (1)] [5]–[8]. However, in none of these cases have subsequent, additional adaptive mutations been detected, posing the question of whether selection of second-step adaptive mutations is possible or necessary for these viruses to persist in nature. This information is critical for understanding and predicting the long-term consequences of pathogen emergence and maintenance in affected areas, which in turn could influence the development and success of targeted intervention strategies for managing outbreaks.

A new lineage of Chikungunya virus (CHIKV) [arbovirus in family *Alphavirus,* genus *Togaviridae*] emerged in 2004 in Kenya and subsequently spread into many countries in the Indian Ocean basin [hence the name: Indian Ocean lineage (IOL)], causing devastating outbreaks of arthritic disease [9]. In India, IOL strains were first detected in December 2005 followed by extensive geographic expansion during 2006–2011 into 19 Indian states with a total number of human cases estimated in 2007 at between 1.4 and 6.5 million [10], [11]. During 2006, the states most affected by CHIKV were Karnataka and Maharashtra, with a subsequent shift to Kerala, Coastal Karnataka and West Bengal [12], [13]. Several hypothetical factors may have contributed to the CHIKV emergence/spread on the Indian subcontinent [14], including: 1) the use of immunologically naïve human populations for maintenance, amplification and virus dispersal among localities, 2) reliance on peridomestic and anthropophilic mosquitoes as vectors, and 3) the IOL-specific genetic predisposition for rapid adaptation to *Aedes* (*A.*) *albopictus*, which was previously considered only a secondary CHIKV vector [9].

The mode of CHIKV maintenance in nature is complex and appears to be region-specific. In Africa, CHIKV is maintained in enzootic cycles involving transmission between non-human primates and canopy-dwelling, primatophilic *Aedes* mosquitoes, primarily *A. furcifer, A. taylori*, *A. africanus*, *A. luteocephalus* and *A. neoafricanus* [15]–[19]. In contrast, CHIKV transmission in Asia is believed to rely on humans alone as reservoir/amplification hosts, with the domestic *A. aegypti* and to lesser extent the peridomestic *A. albopictus* serving as primary urban mosquito vectors [19], [20]. Recent evidence, however, suggests the possibility of additional sylvatic, zoonotic transmission cycles [21], [22].

In India, both urban CHIKV vectors are present, although their distributions differ, and their epidemiologic significance for CHIKV transmission probably varies locally. *A. aegypti* was considered to be the most important during the early phase of the CHIK epidemic in 2006 [23]. However, in subsequent years (2007–2009), the involvement of *A. albopictus* as the principal vector was documented at least in the states of Kerala and Coastal Karnataka [24]–[27]. Interestingly, CHIKV transmission by *A. albopictus* was shown to be associated with the acquisition of the A226V amino acid substitution in the E1 envelope glycoprotein [24], [28]–[32] (Figure S1) that is responsible for alphavirus virion assembly and virus fusion in endosomes of target cells [33]–[35]. The role of the E1-A226V substitution on CHIKV adaptation to *A. albopictus* was directly demonstrated in laboratory studies, including those using reverse genetics, showing that this mutation is directly responsible for increased CHIKV infection, dissemination and transmission by this vector species [6], [36]. In India, evidence that CHIKV was undergoing genetic adaptation to *A. albopictus* via the E1-A226V substitution first came from Kerala State. During 2006, only the E1-226A variant was recovered there; however, during subsequent years (2007–2008), all isolates sequenced possessed the E1-226V residue [24] (Figure S1). In 2008 the E1-A226V substitution was also found among the majority of CHIKV isolates from Coastal Karnataka, adjacent to Kerala [37], suggesting introduction from the latter state.

In a follow-up study conducted in the state of Kerala, a novel substitution in the E2 envelope glycoprotein, L210Q, was discovered in all human and mosquito CHIKV isolates collected during 2009 [27] (Figure S1). The E2 protein is located on the tips of alphavirus spikes and interacts with host cell receptors as well as with neutralizing antibodies [38], [39]. The L210Q substitution has not been reported in any other CHIKV strains, including those isolated in Kerala State during 2006–2008. This suggests that E2-L210Q substitution was selected as a result of CHIKV adaptation to specific ecological conditions present in Kerala State. Position E2-210 is located in the domain B of the E2 glycoprotein [39], and several earlier studies demonstrated that mutations in this domain mediate host specificity of several alphaviruses [5], [7], [40]–[42] as well as the selection of escape mutants by neutralizing antibodies [43]–[45]. Moreover, we recently demonstrated that epistatic interactions between mutations at positions E1-226 and E2-211 of CHIKV influence the penetrance of the E1-226V substitution for fitness in *A. albopictus* [46]. The E2-I211T substitution was probably acquired by IOL CHIKV strains around 2004–2005 [47], and provides a suitable background to allow CHIKV adaptation to *A. albopictus* via the subsequent E1-A226V substitution.

Considering that *A. albopictus* was a principal CHIKV vector in the state of Kerala in 2009, it was hypothesized that the novel substitution E2-L210Q provided an additional selective advantage for CHIKV transmission by this mosquito [27]. To test this hypothesis we undertook a comprehensive reverse genetic analysis of the effects of E2-L210Q in various CHIKV hosts. Our observations demonstrate that the E2-L210Q substitution mediates a significant increase in CHIKV dissemination in *A. albopictus* by increasing initial infectivity for midgut epithelial cells. In addition, we show that the E1-A226V substitution has a significantly stronger effect on CHIKV fitness in *A. albopictus* than E2-L210Q, probably explaining the observed time differences required for selective sweeps of these mutations in nature.

## Results

### The E2-L210Q substitution is responsible for increased CHIKV dissemination in *A. albopictus*

To investigate the effect of the E2-L210Q substitution on CHIKV fitness in *A. albopictus* mosquitoes, we employed a reverse genetics approach based on the SL-CK1 strain of CHIKV (hereafter abbreviated SL07), isolated in 2007 in Sri Lanka [9]. Previous phylogenetic studies indicated that SL07 evolved from the Indian subgroup of IOL and represents one of the most closely related isolates to strains responsible for CHIKV outbreaks in India (including the Kerala state) [9], [48]. The SL07 isolate was passed only twice on Vero cells since its isolation from a febrile patient, thus limiting the potential for cell culture-adaptive mutations that can artificially influence alphavirus fitness in vertebrate and/or mosquito hosts. The SL-07 strain has an alanine residue at E1 position 226 and a leucine residue at E2-210, corresponding to prototype IOL strain introduced into India in late 2005. Since the E2-L210Q substitution was only detected in CHIKV strains form Kerala that had previously acquired the *A. albopictus-*adaptive E1-A226V substitution [24], single E1-A226V and double (E1-A226V and E2-L210Q) substitutions were introduced into an infectious clone (i.c.), generated from the SL07 strain using site-directed mutagenesis. In addition, a clone with the single E1-A226V substitution (SL07-226V) was genetically marked by introducing a synonymous mutation 6454A→C that creates an *Apa*I restrictase site (SL07-226V-Apa). Previously we demonstrated that the 6454A→C substitution does not influence CHIKV fitness *in vitro* or *in vivo* [6]. The infectious viruses SL07-226V-Apa and SL07-226V-210Q were rescued by electroporation of *in vitro*-transcribed RNA into Vero cells. The specific infectivity and viral titers in cell culture supernatants were almost identical for all constructs (Table S1), indicating that the introduced mutations did not attenuate CHIKV in Vero cells.

**Figure 1:**
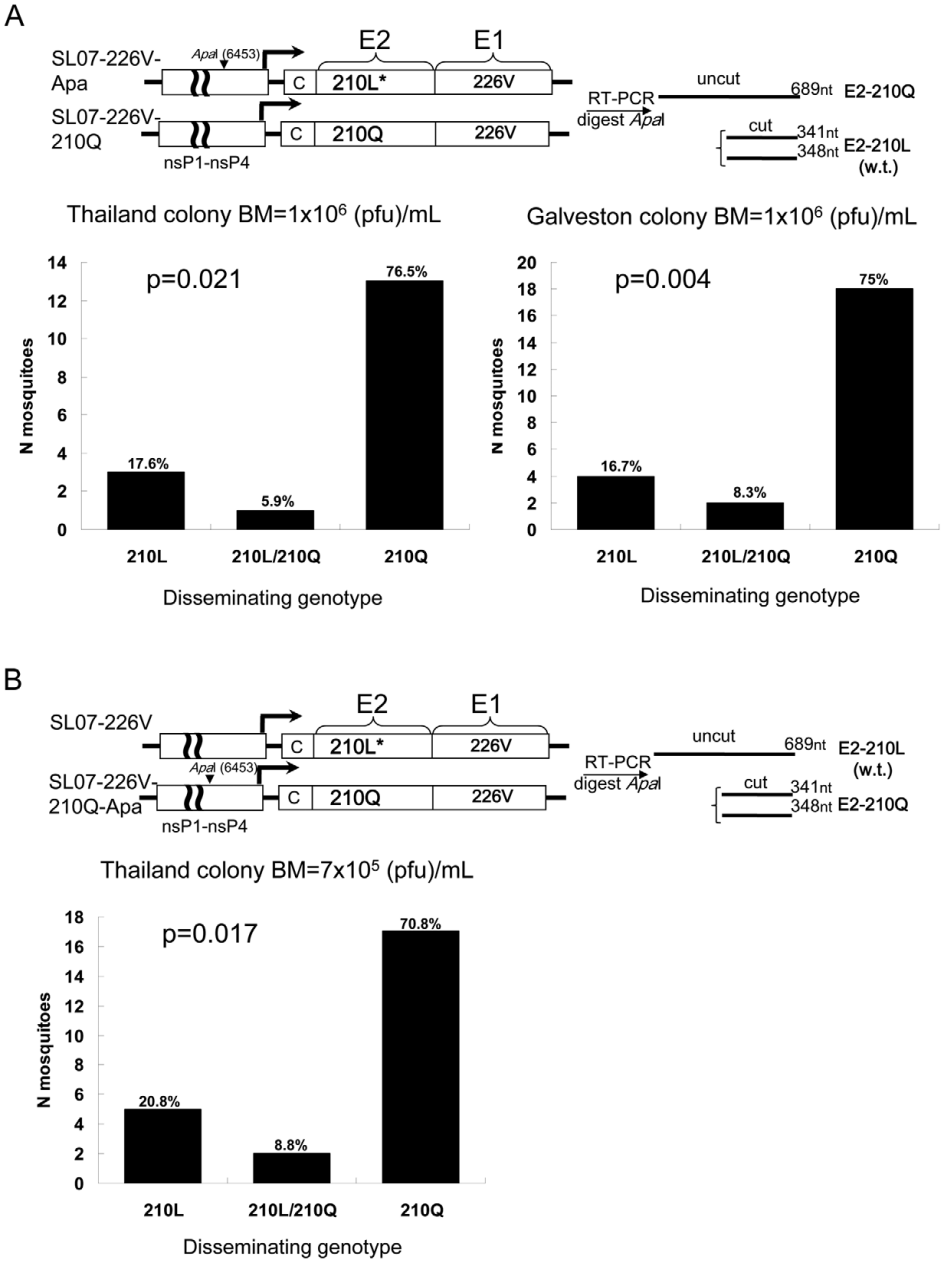
Effect of the E2-L210Q substitution on dissemination of CHIKV in *A. albopictus* mosquitoes (Galveston and Thailand colonies). Above each figure is a schematic representation of the viruses used in the competition assay. Asterisks indicate authentic (w.t.) residues for the SL07 strain at the indicated positions. A 1∶1 mixture of viruses [SL07-226V-Apa and SL07-226V-210Q] (**A**) and [SL07-226V and SL07-226V-210Q-Apa] (**B**) was orally presented to *A. albopictus* and at 10 dpi, the presence of disseminated E2-210L and E2-210Q CHIKV infection was assayed as described in the Materials and Methods. Graphs show numbers and proportions of mosquitoes containing virus populations expressing leucine (210L), glutamine (210Q) or containing both residues (210L/210Q) in mosquitoes heads and legs (representing disseminated infections). The difference in number of mosquitoes with E2-210L versus E2-210Q residues was tested for significance with a one-tailed McNemar test. BM indicates combined titers of competitors in blood meals used for mosquito infection.

Although a variety of mechanisms may be involved, adaptation of arboviruses for enhanced transmission by mosquitoes is typically expected to result in an increased ability to develop a disseminated infection leading to salivary gland infection. To investigate the effect of the E2-L210Q substitution on CHIKV fitness in *A. albopictus* mosquitoes, direct competition experiments were performed using SL07-226V-Apa and SL07-226V-210Q viruses (Figure 1). For these experiments, *A. albopictus* (Thailand strain) was presented with blood meals containing a mix of 5x10^5^ plaque forming units (pfu)/mL of SL07-226V-Apa and 5x10^5^ pfu/mL of SL07-226V-210Q viruses (combined titer 10^6^ pfu/mL) and 10 days post-infection (dpi), the presence of disseminated viral infection as sampled from individual mosquito legs and heads was analyzed as described in the Materials and Methods. The dissemination of the SL07-226V-210Q in the Thailand colony of *A. albopictus* was 4.3 times more efficient compared to SL07-226V-Apa (Figure 1A)(p = 0.021), supporting the hypothesis that glutamine at E2-210 was selected in CHIKV population in Kerala State due to its positive effect on CHIKV transmission. To corroborate these findings, the *Apa*I site was introduced into the backbone of SL07-226V-210Q, and the resultant virus (SL07-226V-210Q-Apa) was tested in direct competition in *A. albopictus* (Thailand colony) against SL07-226V that was produced by reverting the *Apa*I site in SL07-226V-Apa to the wild-type (w.t.) nucleotide sequence (Figure 1B). The dissemination of SL07-226V-210Q-Apa was 3.4 times higher than that of SL07-226V (p = 0.017), indicating that the genetic marker was not responsible for the competition outcome, and supporting the role of the E2-L210Q substitution in increased CHIKV dissemination in *A. albopictus.* To demonstrate that the outcome of competition experiments was not affected by CHIKV propagation in Vero cells (which were used to identify mosquitoes with disseminated infection, prior to CHIKV genotype analysis), these cells were infected at a multiplicity of infection of 0.1 pfu/cell in triplicate with 1∶1 mixtures of viruses that were used in mosquito competition experiments. At 2 dpi, cell culture supernatants were collected for viral RNA extraction and processed as described in the Materials and Methods. No detectable differences in viral fitness (changes in the ratios of the 2 viruses) were observed after Vero cell passage, indicating that E2-L210Q substitution does not affect CHIKV fitness in these cells (Figure S2).

Early studies of CHIKV competence to infect *A. albopictus* demonstrated significant variation in susceptibility among different geographic strains of this mosquito [49]. To demonstrate that the effect of the E2-L210Q substitution on CHIKV dissemination in *A. albopictus* was not limited to a particular geographic strain, we also compared dissemination efficiency of the SL07-226V-Apa versus SL07-226V-210Q viruses in mosquitoes derived from Galveston, USA. Similar to the results with Thailand mosquitoes, the E2-L210Q substitution provided a mean 4.5-fold increase in dissemination efficiency of CHIKV (p = 0.003) (Figure 1A). These data suggest that the E2-L210Q substitution would likely have a similar effect on CHIKV fitness in *A. albopictus* from Kerala State, India, and from other parts of the world.

**Figure 2:**
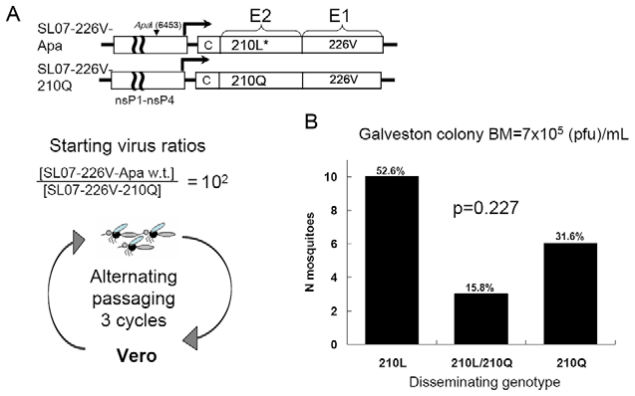
Effect of the E2-L210Q substitution on positive selection of a mutant CHIKV strain within a wild-type population during alternating passaging in *A. albopictus* mosquitoes and Vero cells. **A.** Schematic representation of the alternating passage experiment. The SL07-226V-210Q virus was mixed with 100-fold excess of SL07-226V-Apa virus and presented orally to *A. albopictus* (Galveston). At 10 dpi CHIKV was extracted from combined head and leg homogenates derived from 50 individual mosquitoes and used for Vero cells infection. The cycle was repeated a total of 3 times. At 10 dpi of third mosquito passage, heads and legs of individual mosquitoes were processed as described in the Materials and Methods. **B.** Graph shows numbers and proportions of mosquitoes containing virus populations expressing leucine (210L), glutamine (210Q) or both residues (210L/210Q) in mosquito heads and legs after the third passage in *A. albopictus* (representing disseminated infections). The original mixture used to initiate the infections was not quantified because the PCR-restriction digest assay cannot detect a minority population present at only 1% frequency.

To investigate if the fitness advantage associated with the E2-L210Q substitution is sufficient for selection of mutant viruses in a w.t. CHIKV population, the SL07-226V-210Q was serially passaged in the presence of a 100-fold excess of SL07-226V-Apa (surrogate “wild-type” virus) in an alternating cycle between *A. albopictus* mosquitoes and Vero cells. To initiate the cycle, *A. albopictus* (Galveston colony) were presented with blood meals containing 5x10^5^ pfu/mL SL07-226V-Apa and 5×10^3^ pfu/mL SL07-226V-210Q viruses (100-fold excess of SL07-226V-Apa). After three consecutive passages, heads and legs of individual mosquitoes were processed as described above to determine if selection of virus with E2-L210Q substitution had occurred (Figure 2A). Despite being present in 100-fold lower quantity in the initial virus population, the SL07-226V-210Q virus alone was detected in 31.6% of mosquitoes after 3 alternating mosquito-Vero passages, whereas SL07-226V-Apa (w.t.) alone was found in 52.6% of mosquitoes, while 15.8% of mosquitoes had both competitors in their heads and legs (Figure 2B) (p = 0.227 one-tailed McNemar test). These data indicate that the E2-L210Q substitution has the potential to be selected in CHIKV populations in locations where *A. albopictus* serves as the primary vector. The 31-fold increase over 3 artificial transmission cycles in the relative frequency of SL07-226V-210Q over its initial ratio in blood meals corresponds to a ca. 3-fold increase per cycle, which is in agreement with fitness advantage of the E2-L210Q substitution observed earlier in direct competition experiments (Figure 1).

### The E2-L210Q substitution does not alter CHIKV fitness in *A. aegypti* or human cells

**Figure 3:**
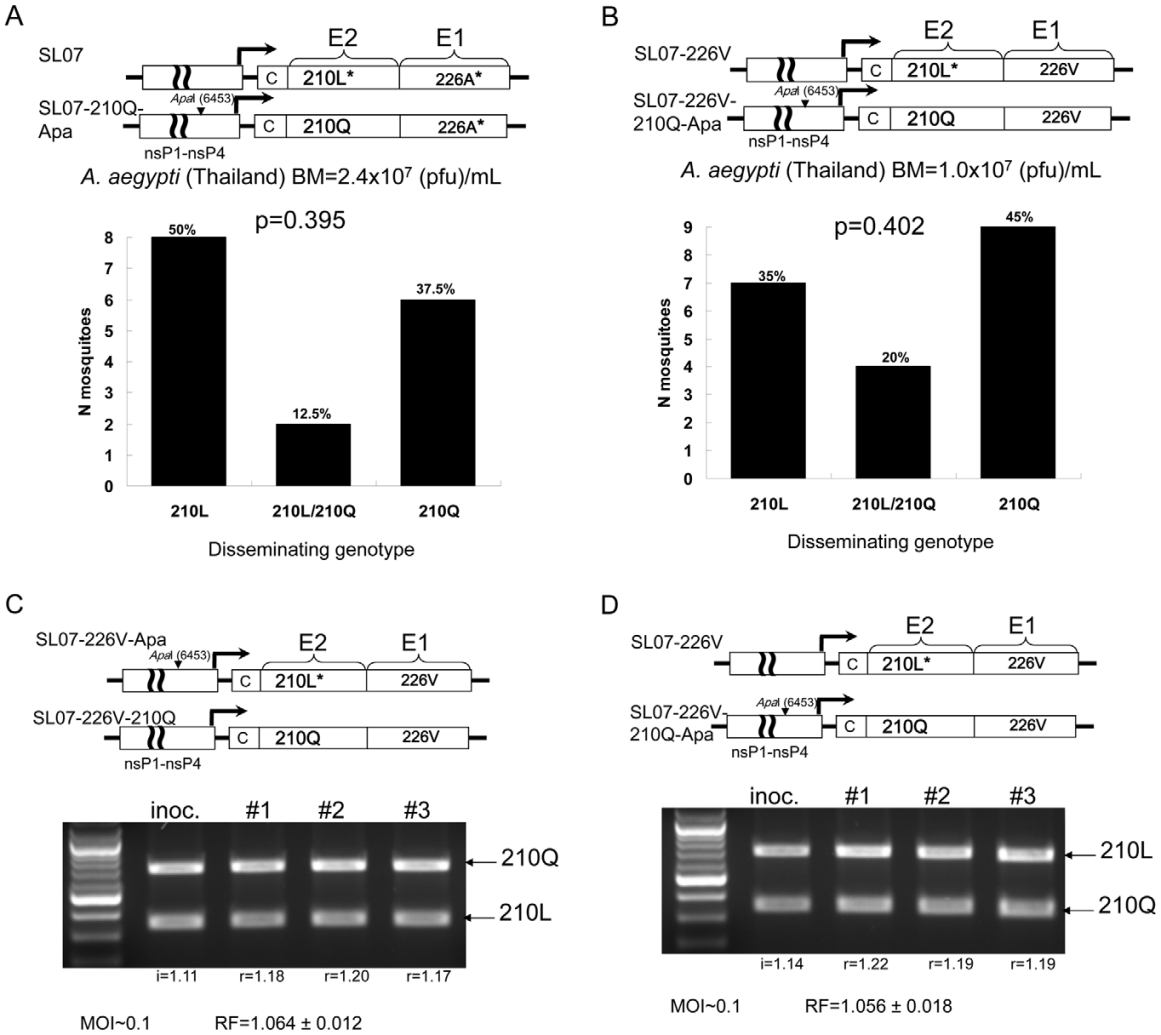
The effect of the E2-L210Q substitution on CHIKV fitness in *A. aegypti* mosquitoes and 293 cells. Above each figure is a schematic representation of the viruses used in the competition assay. Asterisks indicate authentic (w.t.) residues for the SL07 strain at the indicated positions. **A** and **B.** The effect of the E2-L210Q substitution on CHIKV fitness in *A. aegypti*. Graphs show numbers and proportions of mosquitoes containing virus populations expressing leucine (210L), glutamine (210Q) or both residues (210L/210Q) in the background of E1-226A (**A**) and E1-226V (**B**) viruses in heads and legs of *A. aegypti* (Thailand colony) assayed at 10 dpi. BM indicates combined titers of CHIKV (E2-210L and E2-210Q) in blood meals used for mosquito infection. **C** and **D.** The effect of the E2-L210Q substitution on CHIKV fitness in 293 cells. 293 cells were infected at multiplicity of 0.1 pfu/cell in triplicate with 1∶1 mixture of [SL07-226V-Apa and SL07-226V-210Q] (**C**) and [SL07-226V and SL07-226V-210Q-Apa] (**D**). At 2 dpi, supernatants were collected for RNA extraction and RT-PCR analysis. The relative fitness (RF) within a given competition was determined as the average ratio between E2-210L and E2-210Q bands in the sample (r), divided by the starting ratio of E2-210L and E2-210Q bands in the inoculum (i) used for infection.

Historically, *A. aegypti* mosquitoes were the primary vector of CHIKV in Asia [19], [20], and this species still plays a significant role in CHIKV transmission in India [23], [50]–[52]. To investigate if the E2-L210Q substitution also affects CHIKV fitness in *A. aegypti*, we analyzed the effect of E2-L210Q on CHIKV dissemination in this vector using competition experiments as described above. Since CHIKV transmission by *A. aegypti* has never been associated with the E1-A226V substitution, we first used the w.t. genetic background of the SL07 strain (E1-226A and E2-210L) to introduce the E2-L210Q substitution. Also, because *A. aegypti* is less susceptible to CHIKV than *A. albopictus* for strains with E1-226V, we used higher total oral doses up to 2.4×10^7^ pfu/mL (Figure 3A, 3B). The dissemination efficiency of SL07 and SL07-210Q-Apa viruses in *A. aegypti* (Thailand strain) were almost identical (p = 0.395) (Figure 3A). Similarly, no difference in the dissemination efficiency between SL07 and SL07-210Q-Apa viruses was detected in Galveston *A. aegypti* mosquitoes (Figure S3). Additionally, competition between SL07-226V-210Q-Apa and SL07-226V viruses, which express E2-210Q and E2-210L residues in the background of E1-226V, respectively, was also analyzed in Thailand *A. aegypti*, again revealing no statistically significant differences in dissemination efficiency [p = 0.402] (Figure 3B). These data indicate that it is unlikely that the polymorphism at E2-210 affects CHIKV transmission by *A. aegypti*.

Alternatively, E2-L210Q could have been selected as a result of CHIKV adaptation to a vertebrate host in India, probably humans. Although we did not observe any fitness change associated with this mutation during propagation of CHIKV in Vero cells (derived from African green monkey kidneys), to extend our analysis we repeated competition experiments using the human-derived cell line 293 (embryonic human kidney) because earlier studies showed that CHIKV can infect and replicate in various primary human cell lines including epithelial, endothelial, fibroblast, muscle satellite and macrophages (reviewed in [11]). No detectable difference in fitness resulting from the E2-L210Q substitution was observed in this cell culture model (Figure 3B, 3C). Although cell lines are not ideal surrogates for *in vivo* infections, our data further support the conclusion that the E2-L210Q substitution was most likely selected only by *A. albopictus.*

### The E2-L210Q substitution acts by increasing CHIKV infectivity for midgut cells of *A. albopictus*

**Figure 4:**
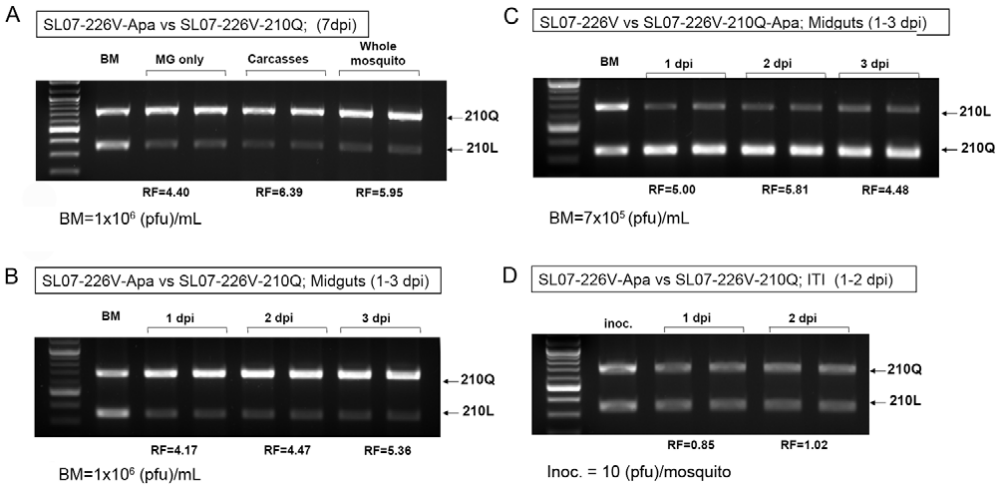
Effect of **the E2-L210Q substitution on CHIKV fitness in** ***A. albopictus*** **bodies, carcasses and midguts after oral or intrathoracic infection.** **A, B** and **C**. *A. albopictus* were fed blood meals containing 1∶1 mixes of [SL07-226V-Apa and SL07-226V-210Q] (**A** and **B**) and [SL07-226V and SL07-226V-210Q-Apa] (**C**) viruses. At 1, 2, 3 (**B** and **C**) and 7 dpi (**A**) whole mosquito bodies (**A**), carcasses without midguts (**A**), or midguts (**A**, **B** and **C**) were collected in pools of ten and processed as described above. BM indicates combined titers of competitors in blood meals used for mosquito infection. **D**. SL07-226V-Apa and SL07-226V-210Q were mixed at a 1∶1 ratio (total concentration of 5×10^4^ pfu/mL), and 0.5 µL was used to infect *A. albopictus* intrathoracically. At 1 and 2 dpi whole mosquitoes were collected in pools of 5 and processed as described above. The relative fitness (RF) for viruses during competition was determined as the average ratio between E2-210Q and E2-210L bands in the sample, divided by the starting ratio of E2-210Q and E2-210L bands in the BM (**A**, **B** and **C**) or inoculum (**D**) used for mosquito infections.

Previous studies determined that the *A. albopictus*-adaptive E1-A226V substitution acts primarily at the level of midgut infectivity. It was suggested that efficient CHIKV infection of and replication in midgut cells promotes more active CHIKV dissemination and transmission by this vector [6], [36], [46], thus allowing the selection of *A. albopictus*-adapted CHIKV strains in nature. To explore which step during CHIKV infection of *A. albopictus* mosquitoes is affected by the E2-L210Q mutation, we first compared the relative ratios of SL07-226V-Apa and SL07-226V-210Q RNAs in whole mosquitoes, mosquito midguts and mosquito carcasses (bodies without midguts) after oral infection (Figure 4A). We observed a marked increase in the relative amount of E2-210Q RNA in all samples analyzed, including midguts at 7 dpi. Furthermore, similar increases in the relative amount of E2-210Q RNA in mosquito midguts were observed as early as day 1 post infection, regardless of which of the two competitors was marked by the *Apa*I site (Figure 4B, 4C). In contrast, no difference in the relative amounts of E2-210Q versus E2-210L RNAs were observed 2 days after intrathoracic infection of *A. albopictus*, when CHIKV titers peak (Figure 4D). When injected intrathoracically, CHIKV does not require infection of and replication within mosquito midguts to disseminate to other organs and tissues, suggesting that the initial infection/replication of the midgut epithelium is a major site of selection of the E2-L210Q substitution in *A. albopictus.*

**Figure 5:**
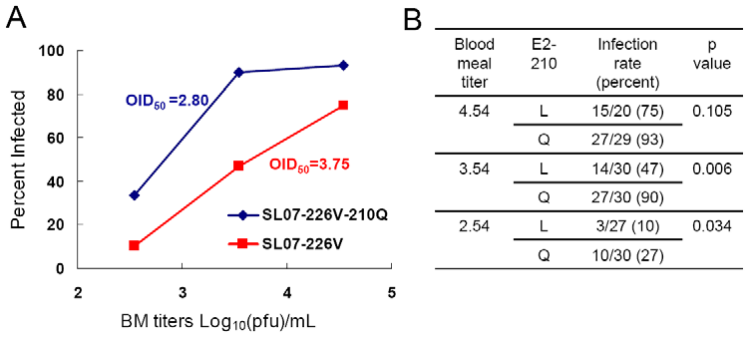
Effect of the E2-L210Q substitution on CHIKV infectivity for *A. albopictus*. Mosquitoes (Thailand) were orally infected with serial 10-fold dilutions of SL07-226V or SL07-226V-210Q viruses in infectious blood meals (BM). At 10 dpi CHIKV infection in individual mosquito was detected by observing virus-induced CPE in Vero cells inoculated with mosquito homogenates. The OID_50_ values were calculated using the PriProbit program (version 1.63) and expressed as Log_10_(pfu)/mL (**A**). The difference in the infection rates between SL07-226V and SL07-226V-210Q viruses was tested for significance with a two-tailed Fishe
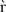
s exact test (**B**).

To further test the hypothesis that the E2-L210Q substitution affects CHIKV fitness only during initial infection of the *A. albopictus* midgut, we first compared infection rates of mosquitoes presented orally with serial dilutions of the viruses expressing either E2-210L or E2-210Q residues in the backbone of the SL07 strain that has the E1-A226V substitution. The E2-210Q residue was associated with significantly higher infectivity (p = 0.006 and p = 0.034, Fishe
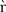
s exact test) for *A. albopictus* (Thailand) at the blood meal titers of 3.5 and 2.5 Log_10_(pfu)/mL, and the oral infectious dose 50% (OID_50_) value calculated for SL07-226V-210Q was 8.9 times lower (higher infectivity) than that for SL07-226V (Figure 5A, 5B). The lack of a significant difference in infection rates after the highest dose (4.54 Log_10_(pfu)/mL) probably reflected the oral dose nearing saturation. By way of comparison, earlier studies, including those using the SL07 strain, determined that the well-established *A. albopictus*-adaptive substitution E1-A226V mediates a much greater, ∼100-fold decrease in OID_50_ values [6], [9].

To directly study the effect of E2-L210Q substitution on initial CHIKV infection of *A. albopictus* midgut cells, we developed a replicon/helper system for the SL07 strain. Sub-genomic replicons of alphaviruses can be packaged into virus-like particles (VLPs) by co-transfection of replicon and helper RNAs into susceptible cells [53]. The helper RNA provides the structural genes that package replicon RNA into VLPs, but the helper RNA itself is not packaged into the VLPs. Therefore, the VLPs are capable of primary infection and replicon RNA replication within cells, but cannot spread to neighboring cells due to the lack of the structural genes in the replicon. Thus, replicon VLPs allowed us to investigate the effect of mutations of interest on initial infection of midgut cells.

Since transfection efficiency of viral RNA is critical in determining the efficiency of VLP production, we switched to BHK-21 cells that have superior RNA susceptibility compared to Vero cells. Earlier, we observed that CHIKV isolates that have not been passaged in rodent-derived cells lines (including SL07) are impaired in their ability to replicate in BHK-21 cells (KT, SCW, unpublished). Therefore, to ensure efficient recovery of CHIKV VLPs from BHK-21 cells, double BHK-adaptive substitutions (nsP1-L407P and nsP3-T348A) were introduced into the SL07 i.c. (see Materials and Methods for details). Although these substitutions increase replication capacity, rather than electroporation efficiency, of CHIKV in BHK-21 cells, they have no effect on mosquito infection (data not shown). The modified SL07 i.c (contains nsP1-L407P and nsP3-T348A substitutions) was subsequently used to generate all CHIKV replicons used in the mosquito infectivity study.

**Figure 6:**
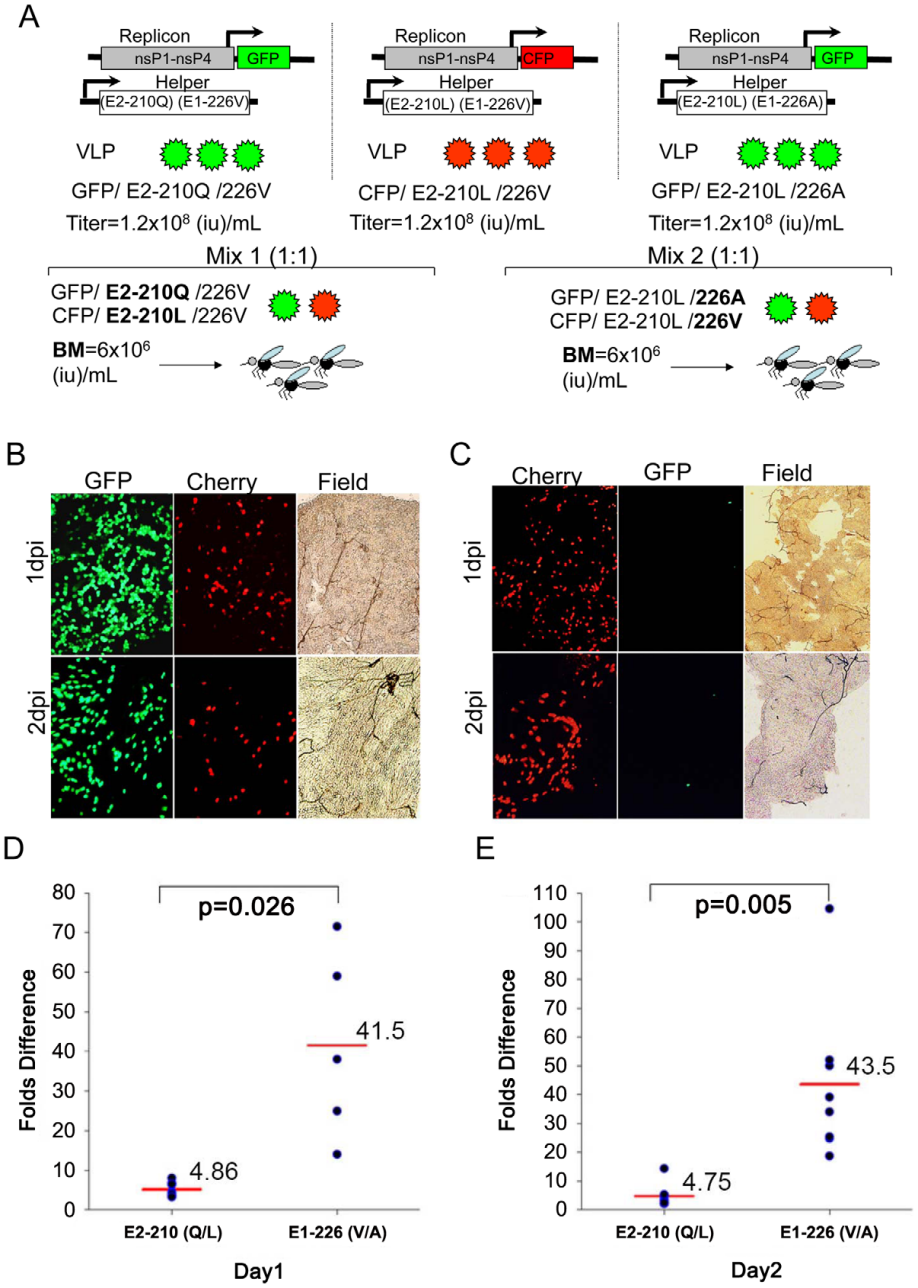
Effect of the E2-L210Q and E1-A226V substitutions on infectivity of CHIKV VLPs for midguts of *A. albopictus* (Thailand). **A**. Schematic representation of VLP production and the experimental design used. At 1 and 2 dpi, mosquito midguts were analyzed by fluorescent microscopy to determine the number of cells expressing GFP and CFP in the same field of vision. **B**. Representative image showing number of GFP- and CFP-expressing cells in individual midguts infected with GFP/210Q/226V and CFP/210L/226V VLPs at 1 and 2 dpi. **C**. Representative image showing number of GFP- and CFP-expressing cells in individual midguts infected with CFP/210L/226V and GFP/210L/226A VLPs at 1 and 2 dpi. **D** and **E**. Each dot corresponds to a fold-difference in the number of cells expressing GFP vs. CFP [E2-210 (Q/L)] or CFP vs. GFP [E1-226 (V/A)] in individual mosquito midguts at 1 dpi (**D**) and 2 dpi (**E**). Red horizontal line represents the mean fold-difference for 5–10 individual midguts. The difference between strains expressing E2-L210Q and E1-A226V residues on VLP infectivity was compared for significance with a two-tailed Studen
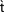
s t-test.

The SL07 replicon expressing green fluorescent protein (GFP) was packaged into VLPs using w.t. SL07 helper (with E2-210L and E1-226A residues) or using a modified helper encoding E2-L210Q and E1-A226V substitutions. The SL07 replicon expressing cherry fluorescent protein (CFP) was packaged into VLPs using a helper encoding E2-210L and E1-226V residues (Figure 6A). In addition, the ApaI marker was introduced into the GFP-expressing replicon. The infectious titers of all recovered VLPs, as determined by titration on Vero cells, were identical (Figure 6A). Infection of Vero and C6/36 cells with 1∶1 mixtures of GFP and CFP expressing VLPs [based on infectious unit (i.u.) titers] yielded equal number of cells expressing these fluorescent proteins (data not shown).

For mosquito experiments, GFP- and CFP-expressing VLPs were mixed 1∶1 (based on i.u. titers) and presented in blood meals to *A. albopictus* as shown in (Figure 6A). At 1 and 2 dpi, midguts of individual mosquitoes were dissected and analyzed by fluorescent microscopy to determine a number of cells expressing GFP and CFP in the same fields of vision (Figure 6B). We found that, on average, midgut cells were 4–5 times more likely to become infected with VLPs expressing the E2-210Q residue as compared with VLPs expressing E2-210L (Figure 6D, 6E). Similarly, 4–5 fold increases in relative amounts of E2-210Q RNA were observed after an *Apa*I digestion of RT-PCR amplicons derived from VLP-infected midguts (Figure S4). Infectious viruses were not recovered after infecting Vero cells with homogenates of 30 mosquitoes infected with VLPs mixes (see Materials and Methods for details), indicating that the hypothetical formation of full-length viruses via recombination between helper and replicon RNAs, which could confound the interpretation of this experiment, did not occur. Altogether, these data demonstrate that the E2-L210Q substitution acts specifically by increasing initial CHIKV infectivity for midgut cells of *A. albopictus*.

In a parallel experiment using VLPs, we also compared the effect of the previously characterized E1-A226V substitution on CHIKV infectivity for midguts of *A. albopictus* (Figure 6A). The CFP-expressing replicon packaged using a helper encoding E2-210L and E1-226V residues (CFP/E2-210L/E1-226V) was competed against GFP-expressing replicon packaged using w.t. SL07 helper encoding E2-210L and E1-226A residues (GFP/E2-210L/E1-226A). In contrast to the polymorphism at E2-210, the valine residue at position E1-226 provided a far greater (41-43 fold) increase in a midgut cell infection compared to the alanine residue at the same position (Figure 6C, 6D, 6E), which agrees with previous results using infectious viruses [6], [36]. These data also indicate that the results of experiments using VLPs with 2 different fluorescent reporter proteins (GFP and CFP) cloned into replicons RNAs are not influenced by those reporter proteins themselves. The significant difference of ∼10-fold between the effects of the polymorphisms at positions E1-226 versus E2-210 on CHIKV infectivity (p = 0.026 and p = 0.005 for 1 and 2 dpi respectively) (Figure 6D, 6C) indicates that the E1-A226V substitution exerts significantly stronger selection compared to E2-L210Q, and thus would be expected to be selected faster during CHIKV transmission by *A. albopictus*.

**Figure 7:**
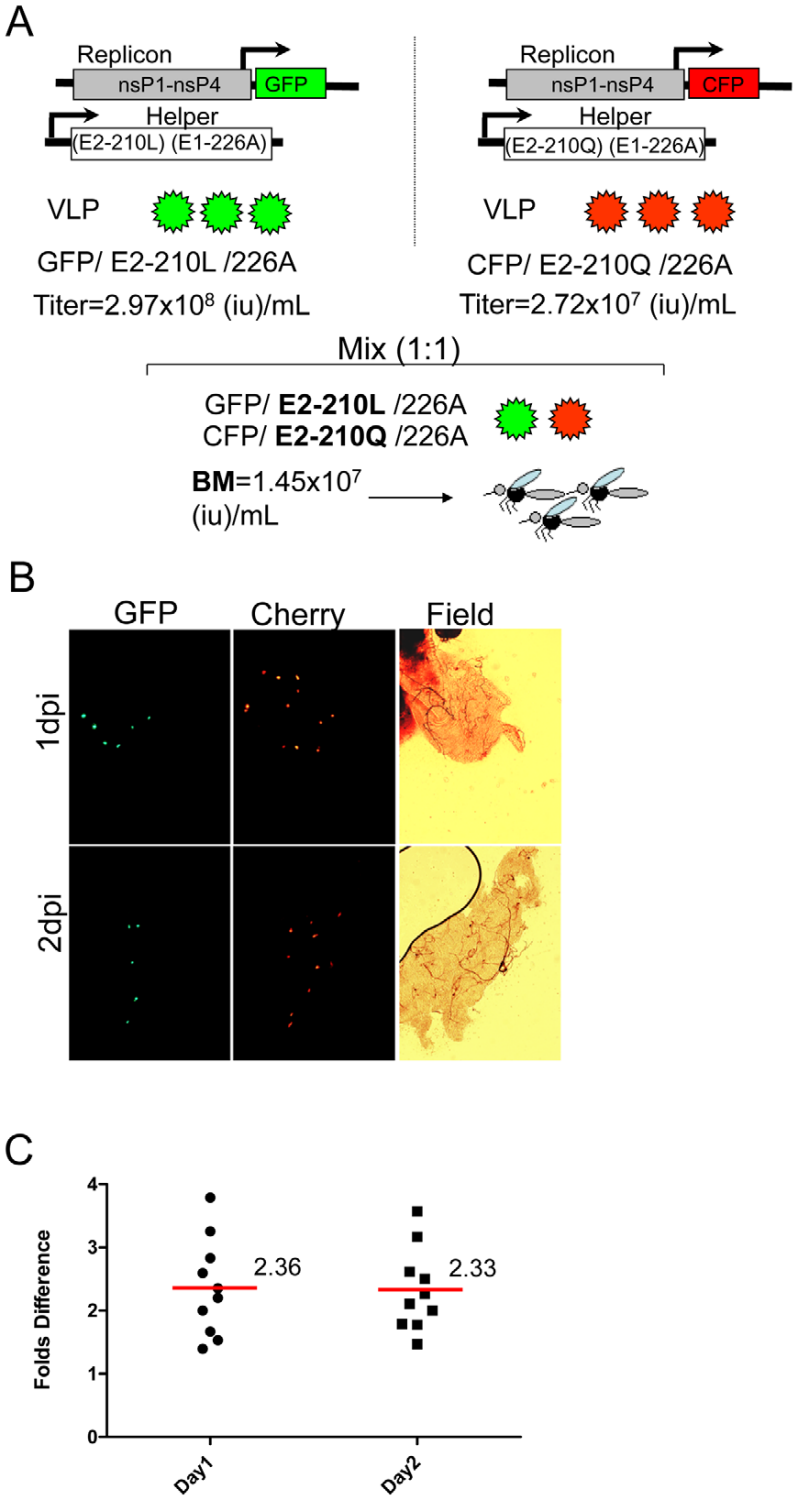
Effect of the E2-L210Q substitution, expressed in the background of E1-226A, on infectivity of CHIKV VLPs for midguts of *A. albopictus* (Thailand). **A**. Schematic representation of VLP production and the experimental design. At 1 and 2 dpi, mosquito midguts were analyzed by fluorescent microscopy to determine the number of cells expressing GFP and CFP in the same field of vision. **B**. Representative image showing number of GFP- and CFP-expressing cells in individual midguts infected with GFP/210L/226V and CFP/210Q/226A VLPs. **C**. Each dot corresponds to the fold-difference in the number of cells expressing CFP vs. GFP [E2-210 (Q/L)] in individual mosquito midguts at 1 dpi and 2 dpi. Red horizontal line represents the mean fold-difference for 10 individual midguts.

To corroborate these findings we also analyzed effect of the E2-L210Q substitution on CHIKV infectivity for midgut cells of *A. albopictus* when this substitution is expressed in the background of w.t. CHIKV with the E1-226A residue. For this experiment, a GFP-expressing replicon was packaged using a w.t. SL07 helper encoding E2-210L and E1-226A residues (GFP/E2-210L/E1-226A), and was competed against a CFP-expressing replicon packaged using a helper encoding E2-210Q and E1-226A residues (GFP/E2-210Q/E1-226A). The E2-L210Q substitution caused a 2.3–2.4-fold increase in CHIKV infectivity for *A. albopictus* midgut cells (Figure 7), which was about 17.5 times weaker than the effect of the E1-A226V substitution in the same genetic background. Similarly, using direct competition experiments between infectious viruses SL07 and SL07-210Q-Apa (both have the E1-226A residue) we observed that the E2-L210Q substitution provided a mean 2.0-fold increase in dissemination efficiency of CHIKV (p = 0.022) (Figure S5) in the Thailand strain of *A. albopictus*. These data indicate that the E2-L210Q substitution would be selected more efficiently in CHIKV strains that previously acquired the E1-226V mutation.

## Discussion

In this study we showed that an E2-L210Q substitution recently identified in CHIKV populations of Kerala State, India, when expressed in the background of the initial adaptive E1-226V substitution, confers a selective advantage by increasing initial infection of *A. albopictus* midgut epithelial cells. Efficient infection of midguts promotes subsequent CHIKV dissemination into the hemocoel and transmission by this vector. However, the E2-L210Q substitution has no apparent effect on CHIKV fitness in the other primary mosquito vector, *A. aegypti,* or on fitness in cell culture models for primate infection (Vero and 293 cells). These results as well as surveillance data indicating that CHIKV was transmitted primarily by *A. albopictus* in Kerala state of India when the E2-L210Q substitution was first detected [24]–[27], provide a comprehensive evolutionary explanation for its appearance in 2009. These results also indicate that adaptation of CHIKV to *A. albopictus* mosquitoes mediated by the previously characterized E1-A226V substitution was probably just a beginning of multi-step adaptive process that included the selection of a second (E2-L210Q) and possibly additional, future mutational steps by IOL strains now circulating in urban areas. These mutations, which have no deleterious effect on transmission by *A. aegypti*, will enable CHIKV to even more efficiently exploit urban transmission in environments populated by *A. albopictus,* but also to maintain the ability to utilize *A. aegypti*, which tends to occur in major urban centers [54]. Thus, our findings regarding the continued adaptation of CHIKV to *A. albopictus* raise serious public health concerns that even more efficient transmission may exacerbate the already devastating CHIK epidemics in India and Southeast Asia. Furthermore, the introduction of the E2-L210Q strain into new areas like Italy and France, where autochthonous cases have already occurred [55]–[57], could spread epidemics into temperate climates where *A. albopictus* thrives. Considering the broad global distribution of *A. albopictus,* including nearly throughout the Americas, the E2-L210Q substitution may significantly increase the risk of CHIKV becoming endemic in additional locations.

Interestingly, Niyas et al. (2010) demonstrated that CHIKV strains with the E2-L210Q substitution can be isolated from adult *A. albopictus* mosquitoes that were reared from wild-caught larvae collected in Kerala State, suggesting that transovarial transmission (TOT) may also play a role in CHIKV maintenance, especially during dry seasons [27], [58]. Also, evidence suggests that TOT occurred in a small percentage of wild mosquitoes during recent CHIK outbreaks on Reunion Island, Madagascar, and in Thailand [59]–[61]. Although we did not attempt to study the effect of the E2-L210Q substitution on TOT, and at least one laboratory study failed to demonstrate TOT in *A. albopictus* of CHIKV strains with the E1-A226V substitution [58], so the possibility that CHIKV mutations could influence rates of TOT warrants a thorough investigation.

**Figure 8:**
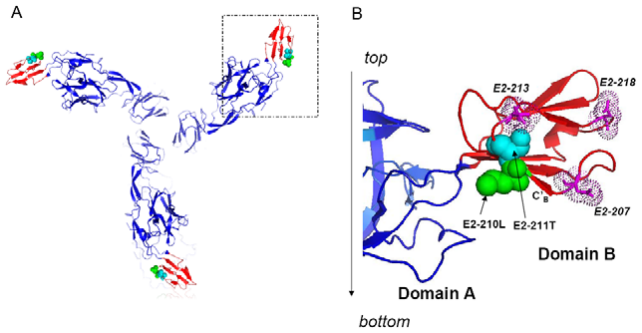
Atomic structure of the CHIKV E2 glycoprotein demonstrating positions in domain B involved in regulation of the alphavirus host range. **A**. Trimeric form of E2 protein, view from the top. **B**. Domain B of CHIKV E2 protein with positions involved in CHIKV adaptation to *A. albopictus* [green (E2-210) and cyan (E2-211)]. Positions involved in modulation of VEEV host range are in magenta [5], [7], [42]. Image is constructed based on atomic structure of CHIKV E2 protein [PDB ID:3N44, [39]]. The 3-D model was analyzed using the PyMol molecular viewer [70].

The molecular mechanism explaining the effects of the E2-L210Q substitution on CHIKV infectivity for *A. albopictus* midgut cells remains unknown. Earlier, we hypothesized that the E2 region around position 211 could be directly involved in interactions with a specific cell surface receptor [46]. We showed that the E2-211T residue mediates a significant increase in infectivity for *A. albopictus* in concert with the E1-A226V substitution, and that residue E2-211I, which is common among CHIKV strains, blocks this effect. Moreover, using virus overlay protein binding assays (VOPBA) to study CHIKV binding to the proteins associated with the brush border membrane fraction of *A. albopictus* midguts, we demonstrated that the E2-T211I substitution dramatically alters CHIKV interactions with as yet unidentified proteins (KT unpublished). The recently determined crystal structure of the CHIKV E2 glycoprotein [39] provides additional insights into the possible involvement of residues E2-211 and E2-210 in interactions with a putative mosquito receptor (Figure 8). Both positions are located at the C'_B_ sheet of the E2 protein, which is exposed on the virion surface on the lateral side of domain B, suggesting that these positions could be involved in interactions with cellular proteins. Substitutions of the aliphatic moieties with polar residues in this region may therefore be directly responsible for changing CHIKV affinities to as yet unidentified receptor(s). Interestingly, positions E2-207, E2-213 and E2-218, which have been shown to be involved in VEEV adaptation to equine and mosquito hosts [5], [7], [42], are also located in the same lateral surface of domain B, further supporting the hypothesis that E2-L210Q enables CHIKV to interact with a particular protein expressed on the surface of midgut cells. The studies to identify these protein(s) are underway.

In the study by Niyas et al (2010) that discovered the E2-L210Q substitution in CHIKV strains from Kerala, only limited portions of CHIKV genomes including the nsP2, E2 and E1 genes were sequenced [27]. Since we did not have an access to these isolates or to complete sequence of these strains, we cannot rule out the possibility that other genome regions could be influencing CHIKV evolution in Kerala State. Epistatic mutations in different genome positions can dramatically affect CHIKV infection of *A. albopictus* [9]. For example, the recently determined, lineage-specific epistatic interactions between positions E1-226 and E1-98 probably limited for at least 60 years the emergence and establishment of new CHIKV strains in Asian regions inhabited by *A. albopictus* [9]. This suggests that Kerala strains of CHIKV might have acquired adaptive substitutions in addition to E2-L210Q that promote efficient transmission in the human-*A. albopictus* cycle, and indicates the need for a more detailed, continuous molecular characterization of CHIKV strains from throughout its distribution.

We also investigated if residue E1-226 has an epistatic effect on amino acid E2-210. The E2-L210Q substitution was detected only in CHIKV strains that already acquired the E1-A226V substitution. We observed that the E2-L210Q substitution mediates a 4–5-fold increase in *A. albopictus* midgut infectivity when expressed in the background of E1-226V, whereas the same substitution caused only a 2.3–2.4-fold increase when expressed in the background of E1-226A (Figure 6 and 7). These results indicate that selection of this mutation would have been even less efficient if it had occurred in a CHIKV strain that did not yet acquire E1-A226V change. Interestingly, our data show that, with regard to CHIKV infectivity of *A. albopictus* midgut cells, E2-L210Q has an approximately 17-fold (E1-226A background) or 10-fold (E1-226V background) weaker effect compared with E1-A226V (Figure 6 and 7). This could explain why E1-A226V was selected convergently by unrelated CHIKV strains on at least 4 well documented occasions, while selection of E2-L210Q has thus far been observed only once in Kerala State (Figure S1). The stronger fitness effect of E1-A226V is consistent with its historically faster selection, which resulted in a selective sweep in parts of the Indian Ocean, India and Southeast Asia, compared with E2-L210Q, which has predominance in only one location. After CHIKV introduction into a region with large *A. albopictus* populations, the E1-A226V substitution has consistently taken about 0.5-1 year to appear [24], [28], whereas the E2-L210Q change was observed after at least 3 years of circulation in Kerala State [27] (Figure S1). More studies are needed to determine the precise dynamics of the selective sweeps associated with both mutations.

Another interesting observation is that both *A. albopictus-*adaptive substitutions exert their effect on CHIKV fitness primarily at the level of midgut infectivity (Figure 4 and 6). The overall increase in the number of midgut cells infected with CHIKV VLPs expressing E2-210Q correlates with the increase in dissemination efficiency observed for infectious viruses. Also, the relative increase in the amount of E2-210Q RNA in midguts infected with VLPs is almost indistinguishable from the relative increase in amount of E2-210Q RNA in midguts exposed to infectious viruses (Figure 4 and S4). Although we did not examine replication in a comprehensive set of mosquito tissues, these results suggest that, after establishing an initial infection from the midgut lumen, the subsequent spread of viruses among neighboring cells is not influenced by position E2-210. Moreover, no differences were observed in CHIKV replication in *A. albopictus* bodies after intrathoracic infection, indicating that replication of CHIKV in secondary mosquito organs also is unaffected by residue E2-210 (Figure 4). Similar observations were provided earlier for position E1-226 [62]. Experimental studies of epizootic versus enzootic VEEV VLP interactions with the epidemic vector, *A. taeniorhynchus,* also indicated that midgut epithelia is the target organ for VEEV adaptation to this vector [63]. These findings suggest that adaptation of alphaviruses to a mosquito vector primarily occurs at the level of midgut infection.

In summary, we demonstrated that adaptation of CHIKV to a new mosquito vector can be a multistep process that, since 2005, has involved at least 2 amino acid substitutions in the envelope glycoproteins. The substitution that provides the strongest selective advantage, E1-A226V, was followed by second adaptive mutation (E2-L210Q) that has resulted in a strain circulating in India with the fittest phenotype detected yet for transmission by *A. albopictus*. We hypothesize that this sequential adaptation will facilitate even more efficient circulation and persistence of the *A. albopictus*-adapted strains in endemic areas and will further increase the risk of expanded and more severe CHIK epidemics in new geographic ranges. This underscores the need for continued surveillance and studies of ongoing CHIKV evolution, as well as the molecular mechanisms that govern CHIKV adaptation to new environments.

## Materials and Methods

### Viruses and plasmids

The SL07 (SL-CK1) strain of CHIKV was isolated in 2007 from a human in Sri-Lanka (GenBank Acc. No. HM045801.1). This strain belongs to Indian subgroup of the IOL [9] and was obtained from the World Reference Center for Emerging Viruses and Arboviruses (WRCEVA) at the University of Texas Medical Branch, Galveston, TX after its generous submission by Aravinda de Silva of the University of North Carolina. Since its isolation the strain was passed twice on Vero cells before being used for i.c. construction. Viral RNA was extracted from lyophilized virus stock using TRIzol reagent (Invitrogen, Carlsbad, CA), reverse-transcribed using Superscript III (Invitrogen, Carlsbad, CA) and cDNA was amplified using Pfu DNA polymerase (Stratagene, La Jolla, CA) and PCR. To assemble the i.c., overlapping RT-PCR amplicons were cloned into modified pSinRep5 vector (Invitrogen) under the control of an SP6 promoter using a strategy described previously for strain LR2006 OPY1 [64]. Point mutations 10670C→T (E1-A226V), 9170T→A (E2-L210Q) and 6454A→C (synonymous, *Apa*I marker) were introduced in various combinations into the i.c. of SL07 using conventional PCR-based cloning methods [65], and the PCR-generated regions were completely sequenced. Plasmids encoding sub-genomic replicons of strain SL07 were generated from the i.c. of the BHK-21 cell-adapted version of this strain [SL07-BHK that contains 1296T→C and 5087A→G (nsP1-L407P and nsP3-T348A) substitutions] which was reported previously [9]. These mutations were identified by electroporation of the SL07 i.c. into BHK-21 cells, followed by sequencing of the recovered, plaque purified viruses. Replicons were generated by replacing the structural gene region of SL07-BHK with the sequence of eGFP or CFP genes utilizing standard techniques [64], [66]. In addition, a point mutation 6454A→C (synonymous, *Apa*I marker) was introduced into the pRep-GFP construct that allows comparison of the relative RNA quantities in an experimental, mixed infection sample. The helper plasmids were generated by deleting the 373–7270 nt. cDNA fragment from i.c. of SL07 that has mutations of interest at E1-226 and E2-210. Plasmids were propagated using the MC1061 strain of *E. coli* in 2xYT medium and purified by centrifugation in cesium chloride gradients. Detailed information for all plasmids is available from the authors upon request.

### Cells and mosquitoes

Vero cells (African green monkey kidney) were propagated at 37°C, with 5% CO_2_, in Minimal Essential Medium (MEM; Invitrogen, Carlsbad, CA) supplemented with 5% fetal bovine serum (FBS). BHK-21(S) [Baby Hamsters Kidney] and 293 (Human Embryonic Kidney) cells were maintained at 37°C with 5% CO_2_ in MEM-alpha (Invitrogen) supplemented with 10% FBS and 1x MEM vitamin solution (Invitrogen). The Galveston colonies of *A. albopictus* and *A. aegypti* mosquitoes were established from the mosquitoes collected in Galveston, TX (USA). Thailand colonies of *A. albopictus* and *A. aegypti* mosquitoes were established from mosquito eggs collected in Bangkok, Thailand. All manipulations and handling of mosquitoes were done as described previously [67].

### Recovery of the infectious viruses and VLPs from the i.c

Infectious viruses were generated by electroporation of the *in-vitro* transcribed RNA into Vero cells. RNA was transcribed from SP6 promoter of the *Not*I linearized i.c. DNA using the mMESSAGE mMACHINE kit (Ambion, Austin TX). Ten µg of RNA were electroporated into 10^7^ Vero cells using a BTX-Harvard Apparatus ECM 830 Square Wave Electroporator (Harvard Apparatus, Holliston, MS) and 2mm cuvette at the following conditions: 680V, pulse length 99 µs, 5 pulses, with an interval between the pulses of 200ms. Cells were transferred to a 75 cm^2^ tissue culture flasks with 14 mL of Leibovitz L-15 (L-15) medium supplemented with 10% FBS and 5% tryptose phosphate broth (Sigma-Aldrich, St. Louis, MO). At 3 h post electroporation the cell supernatant was replaced with 14 mL of L-15 medium and maintained at 37 °C without CO_2_. Cell culture supernatants were collected at 24 and 48 h and stored at −80°C.

To estimate the specific infectivity of electroporated RNAs, an aliquot containing 1x10^5^ electroporated Vero cells was serially ten-fold diluted and cells were allowed to attach to sub-confluent monolayers (1x10^6^ cells/well) of uninfected Vero cells in six-well plates [64]. After 2 h of incubation at 37°C, cells were overlaid with 0.5% agarose in MEM supplemented with 3.3% FBS and incubated for 48 h until plaques developed. The results (specific infectivity values) were expressed as pfu/µg of electroporated RNA (Table S1). Titers of the viruses recovered after electroporation and all experimental samples were determined by titration on Vero cells by plaque assay as previously described [7].

To generate CHIKV VLPs expressing residues of interest in E2 and E1 glycoproteins, BHK-21(S) cells were used, which have superior RNA susceptibility compared to Vero cells. To ensure efficient recovery of CHIKV VLPs from BHK-21 cells, all CHIKV replicons were designed to include BHK-adaptive mutations (nsP1-L407P and nsP3-T348A) identified after rescue of w.t. i.c's in BHK-21(S) cells. Ten micrograms of *in-vitro* transcribed replicon and helper RNA were mixed and electroporated into 10^7^ BHK-21(S) cells as described above for Vero cells. Cells were maintained in L-15 medium at 37°C, followed by harvesting supernatants at 30 h post-electroporation. The titer of VLPs was determined by titration on Vero cells as described earlier [68]. Briefly, 1×10^6^ Vero cells were seeded in six-well plates and, after a 16 h incubation at 37°C, monolayers were infected with 10-fold dilutions of the samples for 1 h at 37°C, followed by adding 2 mL of MEM. After 24 h of incubation at 37°C the numbers of GFP- or CFP-expressing cells were quantified by fluorescent microscopy and titers were expressed as infectious units (i.u.)/mL.

### Mosquito infectivity experiments

The role of viral mutations at position E2-210 on CHIKV dissemination in *A. albopictus* and *A. aegypti* mosquitoes was analyzed using direct competition experiment as described earlier [6], [9]. A pair of viruses that differed by mutations of interest in the E2 protein was mixed at a 1∶1 ratio, with one of the viruses containing the *Apa*I marker. Viral mixes were used to prepare infectious blood meals by dilution in an equal volume of the defibrinated sheep blood (Colorado Serum, Denver, CO), then orally presented to 4–5 day old female mosquitoes at 37°C as described previously [6], [67]. Ten days post infection, heads and legs of individual mosquitoes were triturated in 500 µL of MEM media containing 5 µg/mL of Amphotericin B (Fungizone), and 100 µL of clarified supernatant were added to duplicate wells of a 96-well plate containing 5x10^4^ Vero cells/well. At 3 dpi, supernatant from virus-induced CPE (cytopathic effect)-positive wells was used for RNA extraction followed by RT-PCR with 41855ns-F5 (5̀-ATATCTAGACATGGTGGAC) and 41855ns-R1 (5̀-TATCAAAGGAGGCTATGTC) primers sets using One-Step RT-PCR kit (Qiagen, Valencia, CA). The PCR products were digested with *Apa*I restrictase (NEB, Ipswich, MA) and separated on 1.5% agarose gels followed by ethidium bromide staining. One PCR band in the digested sample corresponded to disseminated infection for one out of two viruses in the pair; two bands indicated that both viruses disseminated in the same mosquito. Differences in dissemination efficiencies were tested for significance with a one-tailed McNemar test.

Viral competition experiments with serial, alternating CHIKV passaging in *A. albopictus* and Vero cells were performed as described above with minor modifications. For the first passage, virus SL07-226V-210Q was mixed with 100-fold excess SL07-226V-Apa to generate infectious blood meals containing 5x10^5^ pfu/mL (combined). The blood meal was used for oral infection of *A. albopictus* (Galveston colony) followed by virus extraction from combined head and leg homogenates derived from 50 individual mosquitoes in 1.5 mL of MEM medium at 10 dpi. Homogenates were filtered and used to infect 75 cm^2^ flasks of Vero cells. At 2 dpi, cell culture supernatants were diluted 1∶10 in L-15 medium and mixed with equal volumes of defibrinated sheep blood to prepare a blood meal for the second passage. The cycle was repeated a total of 3 times. At 10 dpi of third mosquito passage, heads and legs of individual mosquitoes were processed as described above.

For CHIKV competition experiments in specific body parts of *A. albopictus,* the mosquitoes were exposed to blood meals containing 1∶1 mixes of [SL07-226V-Apa and SL07-226V-210Q] and [SL07-226V and SL07-226V-210Q-Apa]. Depending on the experiment, at 1, 2, 3 and 7 dpi whole mosquito bodies, mosquito carcasses, or mosquito midguts were collected in pools of ten, and were used for RNA extraction using TRIzol (Invitrogen, Carlsbad, CA). RNA was RT-PCR amplified, followed by *Apa*I restrictase digestion of amplicons as described above. Gel images were analyzed using TolaLab (version 2.01) and relative fitness for a given virus during competition was determined as the ratio between E2-210L and E2-210Q bands in the sample, divided by the starting ratio of E2-210L and E2-210Q in the blood meal. The results were expressed as an average value of 2 pools of 10 mosquitoes midguts per pool.

For CHIKV competition experiments in intrathoracically infected mosquitoes, 5 pfu of SL07-226V-Apa and 5 pfu of SL07-226V-210Q in 0.5 µL of L-15 media were directly injected into thoraxes of cold-anesthetized *A. albopictus* (Galveston colony) using capillary needles as described previously [69]. RNA from 2 pools, 5 mosquitoes/pool, was extracted at 1 and 2 dpi and processed as described above.

To investigate the relationship between the blood meal titers and infection rates in *A. albopictus,* the SL07-226V and SL07-226V-210Q viruses individually were serially 10-fold diluted, mixed with defibrinated sheep blood and presented orally to *A. albopictus* (Thailand). At 10 dpi individual mosquitoes were triturated in one mL of MEM and used to infect 5x10^4^ Vero cells in duplicate in 96 well plates. CHIKV was detected by observing virus-induced CPE. The difference in the infection rates between SL07-226V and SL07-226V-210Q was tested for significance with a two-tailed Fishe
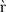
s exact test. The oral infectious dose 50% (OID_50_) values were calculated using the PriProbit program (version 1.63).

For VLP experiments, *A. albopictus* (Thailand) were infected with 1∶1 mixes (based on i.u. titers) of GFP- or CFP-expressing subgenomic replicons packaged into VLPs using CHIKV helpers that differed by substitutions at positions E1-226 and E2-210 (Figure 6A and 7A). At 1 and 2 dpi, 5–10 mosquito midguts were dissected in PBS, and cut longitudinally to generate monolayers of epithelial cells. These sheets were rinsed in PBS to remove residual blood and gently spread out on a glass slide. A cover slip was applied and the midgut sheets were immediately analyzed by fluorescent microscopy to determine the numbers of cells expressing GFP and CFP in the same field of vision. One or two fields of vision were analyzed for each midgut sheet. In parallel experiment, midguts infected with VLPs packaged using helpers that differ by substitutions at position E2-210 were dissected at 1, 2 and 3 dpi, collected in pools of ten, which were used for RNA extraction using TRIzol (Invitrogen). The RNA was processed as described above.

To demonstrate that replicon and helper RNAs did not recombine to generate infectious virus capable of autonomous replication, 30 mosquitoes were infected with VLPs mixes and at 7 dpi were triturated in 1 mL of MEM, filter sterilized and 300 µL of homogenate was used to infect each of 3 wells of confluent Vero cells in six-well plates. After 1 h of infection at 37°C, 2 mL of MEM was added to each well, followed by incubation at 37°C with 5% CO_2_. Cells were observed daily for signs of CPE for 5 days.

### CHIKV competition in Vero and 293 cells

To investigate the effect of substitutions at E2-210 on CHIKV fitness in Vero and 293 cells, these cells were infected at a multiplicity 0.1 pfu/cell in triplicate with 1∶1 mixtures of [SL07-226V-Apa and SL07-226V-210Q] and [SL07-226V and SL07-226V-210Q-Apa] viruses. Cells were maintained at 37 °C with 5% CO_2_ in MEM and at 2 dpi, supernatants were collected for RNA extraction and processed as described above.

## Supporting Information

Figure S1 **Evolutionary history of the E1-A226V and E2-L210Q substitutions in different CHIKV lineages of the ECSA clade.** Black arrows correspond to the emergence and movement of the CHIKV lineages with the E1-226A residue. Red arrows correspond to the acquisition of the E1-A226V substitution. Blue arrow corresponds to acquisition of the E2-L210 substitution. The graph was constructed based on the data published in [24], [27]–[30].
(TIF)

Figure S2 **The effect of the E2-L210Q substitution on CHIKV fitness in Vero cells.** Above each figure is a schematic representation of the viruses used in the competition assay. Vero cells were infected at multiplicity of infection of ∼0.1 pfu/cell in triplicate with a 1∶1 mixture of [SL07-226V-Apa and SL07-226V-210Q] **(A)** and [SL07-226V and SL07-226V-210Q-Apa] **(B)**. At 2 dpi cell culture supernatants were collected for RNA extraction and viral RT-PCR analysis. The relative fitness (RF) within a given competition was determined as the average ratio between E2-210L and E2-210Q bands in the sample (r), divided by the starting ratio of E2-210L and E2-210Q bands in the inoculum (i) used for infection.
(TIF)

Figure S3 **The effect of the E2-L210Q substitution on CHIKV fitness in** ***A. aegypti*** **(Galveston colony).** Above is a schematic representation of the viruses used in the competition assay. Asterisks indicate authentic (w.t.) residues for the SL07 strain at the indicated positions. Graph shows numbers and proportions of mosquitoes containing virus populations expressing leucine (210L), glutamine (210Q) or a mixture of both residues (210L/210Q) in heads and legs of *A. aegypti* (Galveston colony) assayed at 10 dpi. BM indicates combined titers of CHIKV (E2-210L and E2-210Q) in blood meals used for mosquito infection. The difference in number of mosquitoes with E2-210L versus E2-210Q residues was tested for significance with a one-tailed McNemar test.
(TIF)

Figure S4 **Effect of the E2-L210Q substitution on replication of CHIKV replicon particles in A. albopictus midguts after oral infection with VLPs. A** - schematic representation of VLP production and the experimental design used in the mosquito infectivity study. *A. albopictus* (Thailand colony) were orally infected with blood meals containing 3x10^6^ i.u./mL of GFP/210Q/226V and 3x10^6^ i.u./mL of CFP/210L/226V VLPs. At 1, 2 and 3 dpi, mosquito midguts were dissected and 2 pools of 5 midguts per pool were used for RNA extraction and RT-PCR analysis (**B**). Relative fitness (RF) was determined as the average ratio between bands corresponding to VLPs expressing E2-210Q and E2-210L residues in the sample, divided by the initial ratio of E2-210Q and E2-210L bands in the BM used for mosquito infections.
(TIF)

Figure S5 **Effect of the E2-L210Q substitution expressed in the background of the E1-226A residue on dissemination of CHIKV in** ***A. albopictus*** **mosquitoes (Thailand).** Above is a schematic representation of the viruses used in the competition assay. Asterisks indicate authentic (w.t.) residues for the SL07 CHIKV strain at the indicated positions. A 1∶1 mixture of viruses SL07 and SL07-210Q-Apa was presented orally to *A. albopictus* and at 10 dpi, the presence of disseminated E2-210L and E2-210Q CHIKV infection was assayed as described in the Materials and Methods. Graphs show numbers and proportions of mosquitoes containing virus populations expressing leucine (210L), glutamine (210Q) or containing both residues (210L/210Q) in mosquito heads and legs (representing disseminated infections). The difference in numbers of mosquitoes with E2-210L versus E2-210Q residues was tested for significance with a one-tailed McNemar test. BM indicates combined titers of competitors in blood meal used for mosquito infection.
(TIF)

Table S1 **Recovery of the viruses after electroporation of** ***in vitro*** **transcribed RNA.** a – amino acids at positions E1-226. b – amino acids at positions E2-210. c – specific infectivity of *in vitro* transcribed RNA expressed as pfu/1 µgRNA. d – supernatants of electroporated Vero cells were collected at 48 h. Virus titers were determined by titration on Vero cells and expressed as Log_10_(pfu)/mL. h – hours post electroporation.
(DOC)
