# Supplementary figures and images for: Sequential Adaptive Mutations Enhance Efficient Vector Switching by Chikungunya Virus and Its Epidemic Emergence
Source: PLoS Pathog. 2011 Dec 8;7(12):e1002412. doi: 10.1371/journal.ppat.1002412 (PMC3234230; doi:10.1371/journal.ppat.1002412)

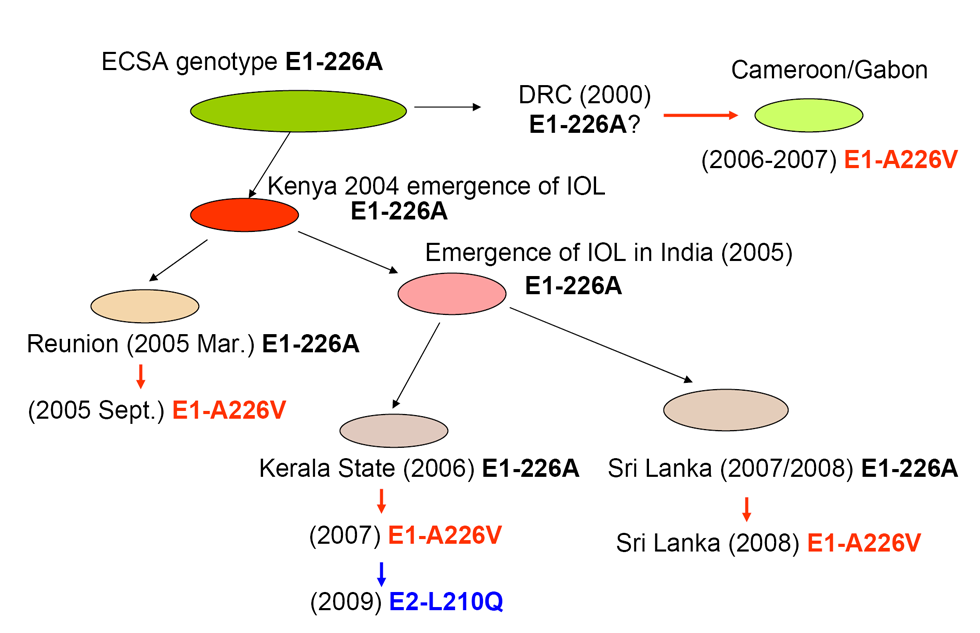

Supplement: Figure S1 — Evolutionary history of the E1-A226V and E2-L210Q substitutions in different CHIKV lineages of the ECSA clade. Black arrows correspond to the emergence and movement of the CHIKV lineages with the E1-226A residue. Red arrows correspond to the acquisition of the E1-A226V substitution. Blue arrow corresponds to acquisition of the E2-L210 substitution. The graph was constructed based on the data published in [24], [27]–[30]. (TIF) [file ppat.1002412.s001.tif]

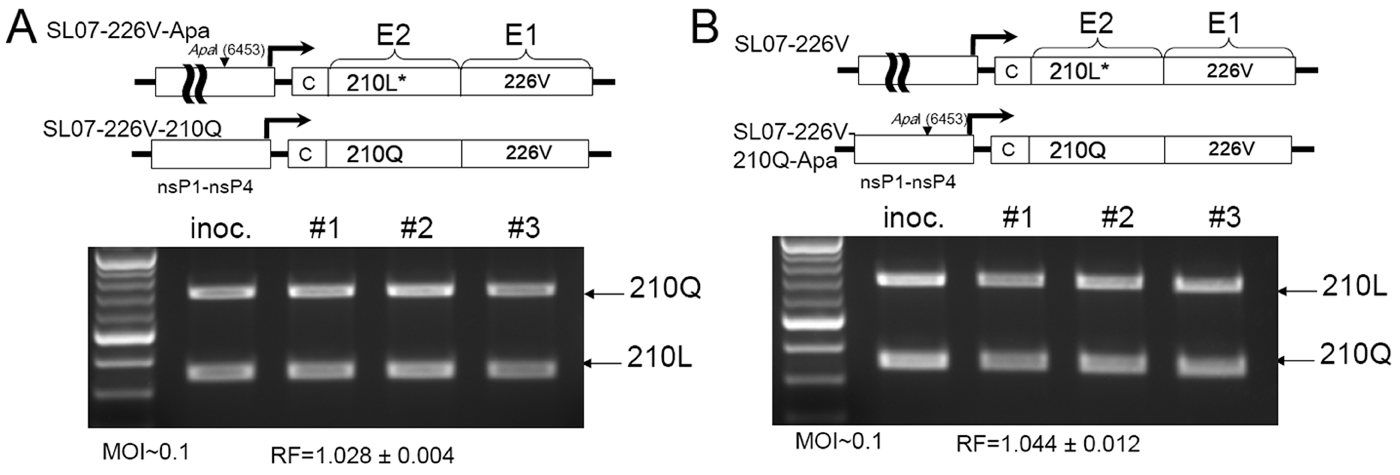

Supplement: Figure S2 — The effect of the E2-L210Q substitution on CHIKV fitness in Vero cells. Above each figure is a schematic representation of the viruses used in the competition assay. Vero cells were infected at multiplicity of infection of ∼0.1 pfu/cell in triplicate with a 1∶1 mixture of [SL07-226V-Apa and SL07-226V-210Q] (A) and [SL07-226V and SL07-226V-210Q-Apa] (B). At 2 dpi cell culture supernatants were collected for RNA extraction and viral RT-PCR analysis. The relative fitness (RF) within a given competition was determined as the average ratio between E2-210L and E2-210Q bands in the sample (r), divided by the starting ratio of E2-210L and E2-210Q bands in the inoculum (i) used for infection. (TIF) [file ppat.1002412.s002.tif]

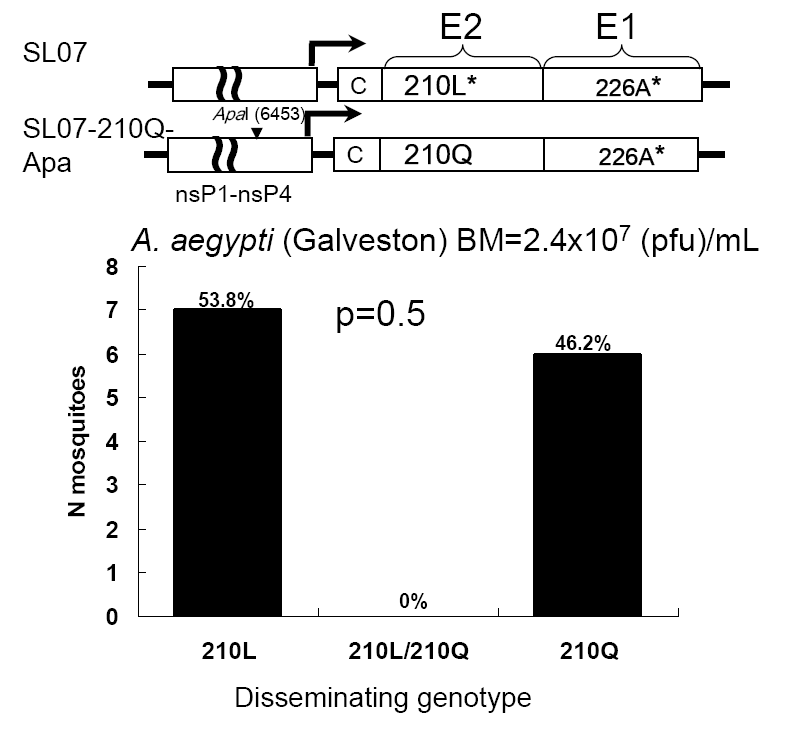

Supplement: Figure S3 — The effect of the E2-L210Q substitution on CHIKV fitness in A. aegypti (Galveston colony). Above is a schematic representation of the viruses used in the competition assay. Asterisks indicate authentic (w.t.) residues for the SL07 strain at the indicated positions. Graph shows numbers and proportions of mosquitoes containing virus populations expressing leucine (210L), glutamine (210Q) or a mixture of both residues (210L/210Q) in heads and legs of A. aegypti (Galveston colony) assayed at 10 dpi. BM indicates combined titers of CHIKV (E2-210L and E2-210Q) in blood meals used for mosquito infection. The difference in number of mosquitoes with E2-210L versus E2-210Q residues was tested for significance with a one-tailed McNemar test. (TIF) [file ppat.1002412.s003.tif]

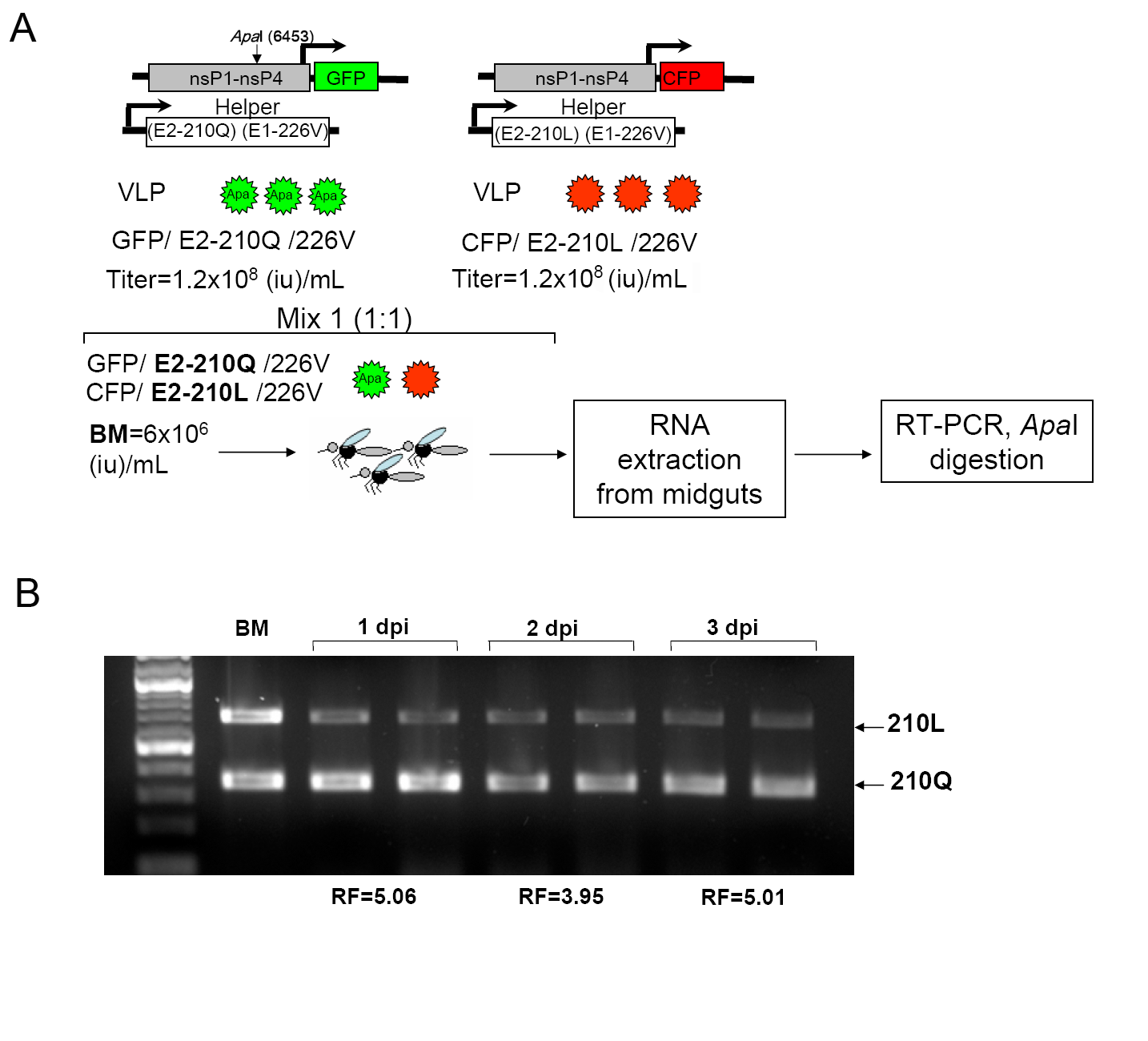

Supplement: Figure S4 — Effect of the E2-L210Q substitution on replication of CHIKV replicon particles in A. albopictus midguts after oral infection with VLPs. A - schematic representation of VLP production and the experimental design used in the mosquito infectivity study. A. albopictus (Thailand colony) were orally infected with blood meals containing 3x10^6^ i.u./mL of GFP/210Q/226V and 3x10^6^ i.u./mL of CFP/210L/226V VLPs. At 1, 2 and 3 dpi, mosquito midguts were dissected and 2 pools of 5 midguts per pool were used for RNA extraction and RT-PCR analysis (B). Relative fitness (RF) was determined as the average ratio between bands corresponding to VLPs expressing E2-210Q and E2-210L residues in the sample, divided by the initial ratio of E2-210Q and E2-210L bands in the BM used for mosquito infections. (TIF) [file ppat.1002412.s004.tif]

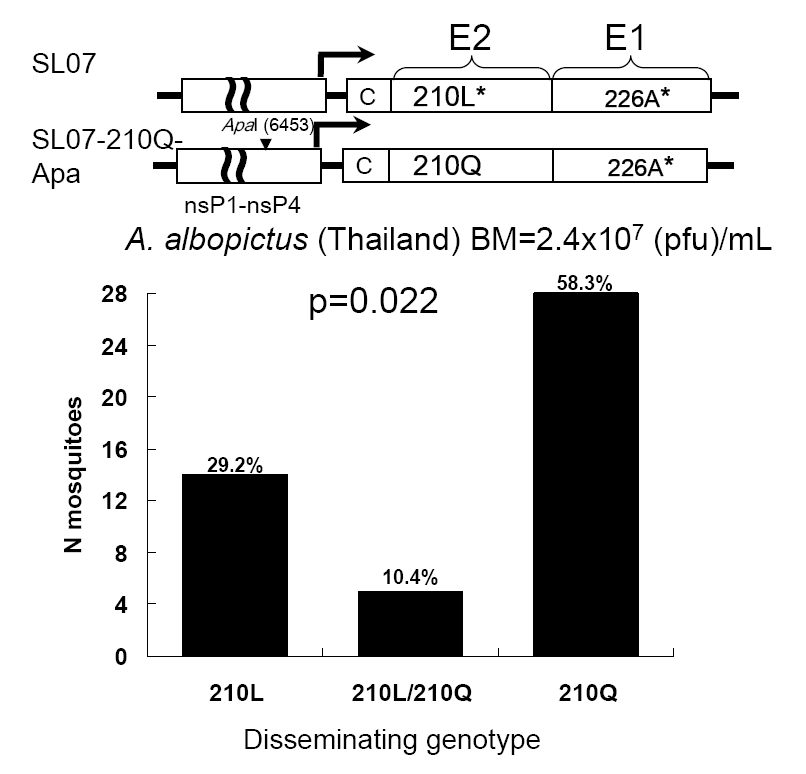

Supplement: Figure S5 — Effect of the E2-L210Q substitution expressed in the background of the E1-226A residue on dissemination of CHIKV in A. albopictus mosquitoes (Thailand). Above is a schematic representation of the viruses used in the competition assay. Asterisks indicate authentic (w.t.) residues for the SL07 CHIKV strain at the indicated positions. A 1∶1 mixture of viruses SL07 and SL07-210Q-Apa was presented orally to A. albopictus and at 10 dpi, the presence of disseminated E2-210L and E2-210Q CHIKV infection was assayed as described in the Materials and Methods. Graphs show numbers and proportions of mosquitoes containing virus populations expressing leucine (210L), glutamine (210Q) or containing both residues (210L/210Q) in mosquito heads and legs (representing disseminated infections). The difference in numbers of mosquitoes with E2-210L versus E2-210Q residues was tested for significance with a one-tailed McNemar test. BM indicates combined titers of competitors in blood meal used for mosquito infection. (TIF) [file ppat.1002412.s005.tif]
