# Supplementary material for: Sequential Adaptive Mutations Enhance Efficient Vector Switching by Chikungunya Virus and Its Epidemic Emergence
Source: PLoS Pathog. 2011 Dec 8;7(12):e1002412. doi: 10.1371/journal.ppat.1002412 (PMC3234230; doi:10.1371/journal.ppat.1002412)
Supplement: Table S1 — Recovery of the viruses after electroporation of in vitro transcribed RNA. a – amino acids at positions E1-226. b – amino acids at positions E2-210. c – specific infectivity of in vitro transcribed RNA expressed as pfu/1 µgRNA. d – supernatants of electroporated Vero cells were collected at 48 h. Virus titers were determined by titration on Vero cells and expressed as Log10(pfu)/mL. h – hours post electroporation. (DOC) [file ppat.1002412.s006.doc]

**Table S1.** Recovery of the viruses after electroporation of *in vitro* transcribed RNA into Vero cells.

| Clone name | E1-226^a^ | E2-210^b^ | Specific infectivity ^c^ | Titer (48 h)^d^ |
| --- | --- | --- | --- | --- |
| SL07 | A | L | 10.0x10^4^ | 5.2x10^7^ |
| SL-226V-Apa | V | L | 7.0x10^4^ | 1.2 x10^7^ |
| SL-226V | V | L | 10.0x10^4^ | 3.45 x10^7^ |
| SL-226V-210Q | V | Q | 7.0x10^4^ | 1.5 x10^7^ |
| SL-226V-210Q-Apa | V | Q | 10.0x10^4^ | 1.5 x10^7^ |
| SL-210Q-Apa | A | L | 10.0x10^4^ | 4.8 x10^7^ |
